# Electrochemically-selective electrode for quantification of dorzolamide in bulk drug substance and dosage form

**DOI:** 10.1186/s13065-023-01021-1

**Published:** 2023-08-21

**Authors:** Mai M. Mora, Nahla S. Ismail, Hala E. Zaazaa, Shereen A. Boltia

**Affiliations:** 1 Egyptian Drug Authority (EDA), Giza, Egypt; 2 Analytical Chemistry Department, Faculty of Pharmacy, Cairo University, Kasr El-Aini St., Cairo, 11562 Egypt

**Keywords:** Dorzolamide, Potentiometric titration, Carbon paste electrode, Silica, β-cyclodextrin

## Abstract

Three smart carbon paste electrodes were fabricated to quantify dorzolamide hydrochloride DRZ, including conventional carbon paste I, modified carbon paste embedding Silica II, and modified carbon paste embedding β-cyclodextrin III. This study is based on the insertion of DRZ with phosphomolybdic acid to create an electroactive moiety dorzolamide-phosphomolybdate ion exchanger using a solvent mediator dibutyl phthalate. The three constructed carbon paste electrodes displayed Nernstian responses and linear concentration ranges with lower detection limits. The vital performance of the created electrodes was verified in relation to various parameters. The electrodes enhance the selective determination of DRZ in the presence of inorganic ions, a co-formulated drug in the dosage form timolol maleate, and the excipient benzalkonium chloride. The modified carbon paste electrode including Silica was utilized to detect DRZ in ophthalmic eye drop form utilizing the direct calibration curve and potentiometric titration methods. Satisfactory findings were achieved by comparing them to other reported methods.

## Introduction

**Fig. 1:**
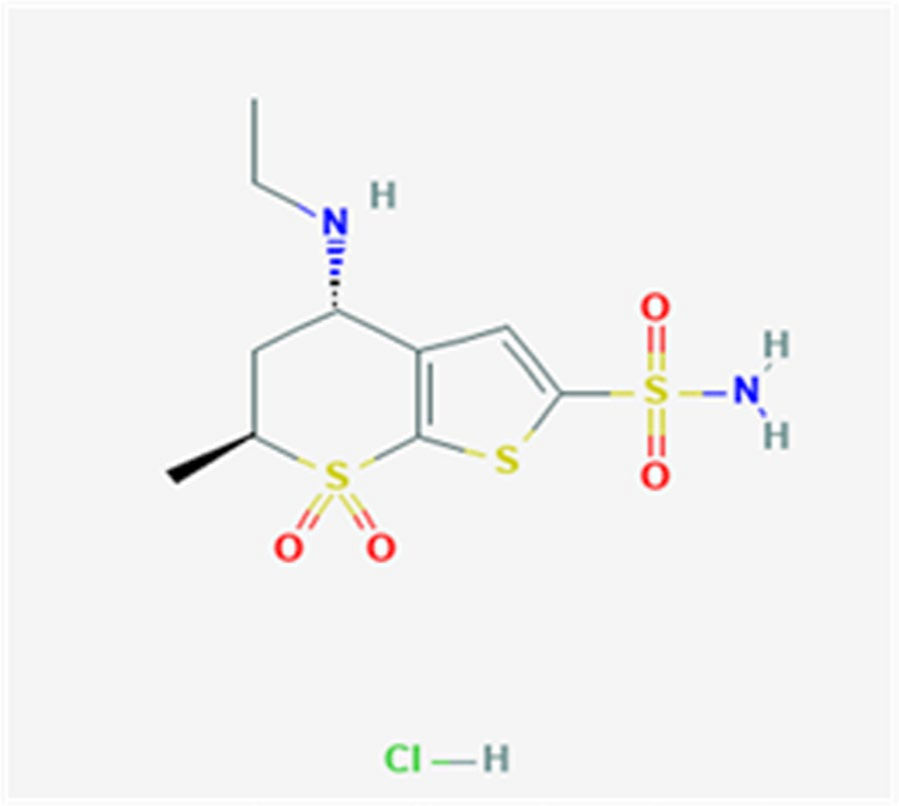
Dorzolamide HCl structure

Dorzolamide HCl Fig. 1 has the IUPAC name of (4 ~ {S},6 ~ {S})-4-(ethylamino)-6-methyl-7,7dioxo-5,6-dihydro-4 ~ H}-thieno[2,3-b] thiopyran-2-sulfonamide; hydrochloride, with a molecular formula of C_10_H_17_ClN_2_O_4_S_3_. Dorzolamide is used in cases with open-angle glaucoma or ocular hypertension to control high intraocular pressure [1]. Moreover, it could be utilized with timolol to indicate patients who are inadequately responsive to ophthalmic beta-blockers [2].

Ion-selective electrodes have been found effective in the analysis of pharmaceutical formulations due to their attractive properties of simple design, ease of construction, reasonable selectivity, fast response time, applicability to colored and turbid solutions, and possible interfacing with automated and computerized systems [3].

The development of chemical sensors has received a widespread attention during the past two decades because of their extensive use in environmental monitoring, clinical analysis, and pharmaceutical formulations via rapid, accurate, reproducible, and low-cost methods. The literature shows that carbon paste electrodes (CPEs) are very useful for a wide range of applications and have thus been devoted to developing new ion-selective electrodes based on carbon paste as the electrode material of choice. Chemically modified have been used as potentiometric sensors in trace analysis for metal ions, organic pollutants, and biological substances. These electrodes are operated by the ion-exchange process of the active component incorporated into the carbon paste matrix [4].

Multiple analytical procedures are reported for the determination of DRZ which are based on different techniques, such as UV spectrophotometry [5–8], HPLC [9–13], and TLC [14]. These reported methods have excellent detection limits and selectivity. Indeed, the study acknowledges that existing methods for the determination of dorzolamide (DRZ) can be costly, require the use of organic solvents, and involve complex apparatus systems, making them impractical for routine analytical assessment. Therefore, there is a need for the development of a selective, accurate, rapid, low-cost, and precise method to detect DRZ.

The goal of this study is to address this need by introducing uncomplicated electrodes with high sensitivity for the determination of dorzolamide. These electrodes are based on modifying traditional carbon paste sensors with β-cyclodextrin and silica. By implementing these modifications, the performance parameters of the electrodes are improved, and their vital parameters are studied for the determination of dorzolamide in both its active pharmaceutical ingredient (API) form and eye drop preparations.

In summary, the study aims to provide a simpler and more cost-effective solution for the detection of dorzolamide by utilizing modified electrodes that offer enhanced sensitivity and improved analytical performance.

## Experimental

### Reagents and chemicals

Dorzolamide HCl drug substance was provided by Precise Chemipharma, Cairo, Egypt, with a purity of 99.55% according to the certificate of analysis. Timolol maleat drug substance was provided by USP with a purity of 99.9%. Xolamol eye drops, produced by Jamjom Pharmaceutical Industries, Jeddah, Saudi Arabia, were purchased from the local market and contained 22.26 mg of Dorzolamide HCl per milliliter (equivalent to 20 mg of dorzolamide) and 6.84 mg of timolol maleate per milliliter (equivalent to 5 mg of timolol). Phosphomolybdic acid (PMA), dibutyl phthalate (DBP), dioctyl phthalate (DOP), *o*-nitrophenyloctyl ether (*o*-NPOE), silica (particle size < 150 μm, 8 nm pore size), glycine amino acid, and aminopencillic acid were received from Sigma-Aldrich (Germany). β-cyclodextrin was obtained from Biochemika Reagent (Germany). Sodium (Na), calcium (Ca), potassium (K), copper (Cu), iron (Fe) working standard solutions of 1000 ppm were purchased from Merck (Germany). Appropriate analytical grade reagents and double distilled water were used throughout the experiments.

### Apparatus

The Ag/AgCl reference sensor was received from Sigma–Aldrich. (St Louis, MO, USA), JENWAY 3510 pH/mV meter with Serial No (06245), made in the UK, was used for potentiometric and pH measurements. Emf measurements using three fabricated carbon paste electrodes were performed using the following electrochemical cell assemblies: AgCl(s),/test solution/working carbon electrode. The electrode performance was studied in a concentration range from 1.0 × 10^–6^ to 1.0 × 1.0^−2^ mol L^−1^ by measuring the emf of DRZ solutions. The solutions were stirred and the emf reading was recorded after the equilibrium of the solution and plotted as a log function of DRZ ion activity.

### Ion-exchanger preparation

To prepare dorzolamide-phosphomolybdate (DRZ-PM), a solution of 100 mL of 10^–2^ mol L^−1^ DRZ was added to 100 mL of PMA (0.0033 mol L^−1^). The resulting precipitates were thoroughly filtered, washed with double distilled water, and dried at approximately 25 °C before being used as the active substance for fabricating the electrodes. The dried precipitates were ground to a fine powder.

### Electrodes fabrication

#### Fabrication of the conventional carbon paste electrode

A conventional carbon paste electrode was simply fabricated by mixing approximately 40% pure micro-sized graphite powder (1–2 μm), 20% DRZ-PM as the electro-active material, and 40% DBP as a liquid plasticizer. The mixture was homogenized to obtain a homogenous paste which was then carefully packed in a Teflon holder (3.0 mm in diameter). The electrode was connected using a copper rod, and its surface was polished with tissue paper to achieve a shiny appearance before use in potentiometric detections. For all potentiometric measurements, the cell assembly consisted of a working carbon paste electrode, the test solution, and an Ag/AgCl reference electrode.

#### Fabrication of the modified carbon paste electrode including silica

Silica-based materials were used as a chemical modifier due to their great surface area and 3D structures, which provide high rates of diffusion for the target drug and many accessible binding sites, resulting in improved electrode sensitivity. To prepare the modified carbon paste electrode including silica, approximately 10% silica, 30% pure carbon powder (1–2 µm), 20% DRZ-PM as the electro-active material, and 40% DBP as a liquid plasticizer were mixed. The subsequent steps were continued following the aforementioned mechanism.

#### Fabrication of the modified carbon paste electrode including β-cyclodextrin

**Fig. 2:**
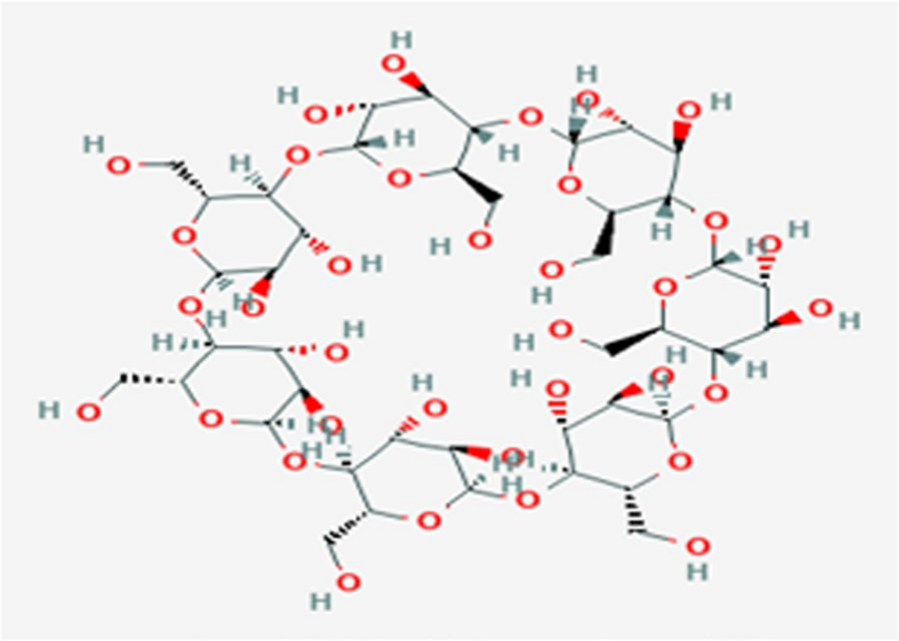
Beta cyclodextrin structure

Cyclodextrins are cyclic oligosaccharides with an external hydrophilic surface and a hydrophobic cavity. They have a high ability to form complexes with different molecules by encapsulating nonpolar analyte molecules within the cyclodextrin cavity. α, β, and γ-CDs are the common types of cyclodextrins, with α-1,4 glycosidic connections and varying numbers of glucopyranosyl units (6, 7, and 8 units, respectively). They have low water solubility because of the strong intermolecular hydrogen bond in the crystal state (Fig. 2). Their solubility is enhanced by substituting the intermolecular hydrogen bond with a hydroxyl group [15]. To fabricate the modified carbon paste electrode including β-cyclodextrin, approximately 10% β-cyclodextrin, 30% pure carbon powder (1–2 µm), 20% DRZ-PM as the electro-active substance, and 40% DBP as the liquid plasticizer were mixed. The subsequent steps followed the aforementioned mechanism.

### Selectivity

The selectivity of an electrode refers to its ability to selectively respond to a specific ion or drug of interest while minimizing the interference from other ions or substances present in the sample. In this context, the selectivity of the ion-selective electrode was evaluated by determining its response to various sugars, organic and inorganic cations, and excipients.

To assess selectivity, the separate solution method (SSM) [16] was employed. The SSM involves two steps:

1. Measurement in a solution with known ion concentration: The electrode potential (emf) is determined using a solution containing a known concentration of the drug (DRZ). This provides a baseline measurement of the electrode's response to the target ion.
2. Measurement in a solution with interfering ions: The emf is measured again, but this time in a solution containing interfering ions at the same concentration. These interfering ions can include common cations, excipients, and amino acids. By comparing the emf values obtained in this step with the baseline measurement, the selectivity coefficient values can be calculated.

The selectivity coefficient (Log K pot DRZ Jz +) is determined using the following formula:

1 $$\mathrm{Log\, K \,pot \,DRZ\, Jz}+=(\mathrm{E}2-\mathrm{E}1)/\mathrm{S}+\mathrm{log}\left(\mathrm{DRZ}\right)-\mathrm{log}(\mathrm{Jz}+)1/\mathrm{z}$$

where E1 is the electrode potential for a 10^–3^ mol L^−1^ solution of DRZ; E2 is the potential of a 10^–3^ mol L^−1^ solution of the interfering cations (Jz+); S is the calibration curve slope; z is the charge of the interfering ion.

If the selectivity coefficient values for the interfering ions are less than 1 compared to the drug (DRZ), it indicates that the interfering ions have a minimal impact on the measured potential. In other words, a lower selectivity coefficient suggests that the ion-selective electrode is more responsive to the target drug and exhibits good selectivity.

### Analytical application of pharmaceutical formulation

#### Direct calibration graph method

Each mL of ophthalmic dosage form batch number (VB0113) contains 22.26 mg of DRZ, so 4.043 mL were taken and dissolved in the least amount of water then this solution was transferred into a 25-mL volumetric flask and sonicate this solution for 5 min, completed to the mark using bi-distilled water to get a final solution of 1 × 10^–2^ mol L^−1^

In the calibration graph method, to a 50 mL volumetric flask, various quantities of DRZ were transferred and completed to volume with water to cover a concentration range from 1.0 × 10^–6^ to 1.0 × 10^−2^ mol L^−1^ and the DRZ-PM/silica electrode was used to measure the emf because this electrode gives the best result of accuracy and precision. Data were plotted with the measured emf against the logarithm of the DRZ concentration and the resulting curve was applied for the following measurement of unknown dorzolamide concentrations in the ophthalmic dosage form.

#### Potentiometric titration method

Aliquots of 3 and 9 mL of 1.0 × 10^–2^ mol L^−1^ DZR solution was added to a 50 mL beaker and was titrated with 0.0033 mol. l-phosphomolybdic acid solution using the modified DRZ-PM with silica as a working electrode. The S-shaped curve was used for the determination of the inflection points.

These analytical methods provide approaches for quantifying the concentration of dorzolamide (DRZ) in pharmaceutical formulations, specifically in ophthalmic dosage forms. The direct calibration graph method utilizes a calibration curve, while the potentiometric titration method relies on the detection of inflection points in a titration curve to determine the concentration of DRZ.

## Results and discussion

### The electrodes composition

The electrode-performing properties depend on the type and amount of the plasticizer used, the nature of the ion exchangers, and their lipophilicity [17, 18]. The effect of paste composition, ion-exchanger amount and nature, and operating conditions on the effective response of the suggested electrode was determined (Table 1). Factors such as response time, interference present, and pH were investigated in relation to the electrode's.

**Table 1:** Optimization of DRZ-PM/silica carbon paste selective electrode and obtained the potentiometric response

No	*I.E	Composition (%)			*LOD mol L^−1^	*RSD	*R (s)
		*C	*P	*S			
1	3 (DRZ-PM)	48.5	48.5 (DBP)	47 ± 0.15	–	1.3	15
2	5 (DRZ-PM)	47.5	47.5 (DBP)	47 ± 0.15	–	1.4	10
3	10 (DRZ-PM)	45	45 (DBP)	48 ± 0.44	–	1.7	10
4	15 (DRZ-PM)	42.5	42.5 (DBP)	50 ± 0.12	–	1.2	9
5	20 (DRZ-PM)	40	40 (DBP)	57 ± 0.1	1 × 10^−4^	0.8	4
6	20 (DRZ-PM)	40	40 (DOP)	10 ± 0.1	–	1.2	9
7	20 (DRZ-PM)	40	40 (DBP)	57 ± 0.1	1 × 10^−4^ mol L^−1^	0.8	4
8	20 (DRZ-PM)	40	40 (NPOE)	43 ± 0.18	1 × 10^−4^ mol L^−1^	1.6	10
9	20 (DRZ-PM)with silica	30	40 (DBP)	59 ± 0.47	1 × 10^−4^ mol L^−1^	0.5	10
	20 (DRZ-PM)with cyclodextrin	30	40 (DBP)	58 ± 0.57	5.96 × 10^−5^ mol L^−1^	0.5	10

*I.E: Ion-exchanger, *C: Carbon (pure carbon)%, *P: % of plasticizer, *S: slope (mVdecade^−1^); *C.R.: concentration range (mol L^−1^), *LOD: detection limit (mol L^−1^); *R (s): response time (s); *RSD: relative standard deviation, measurements replicate number = 3.*Selected composition

#### Ion-exchanger amount effect

**Fig. 3:**
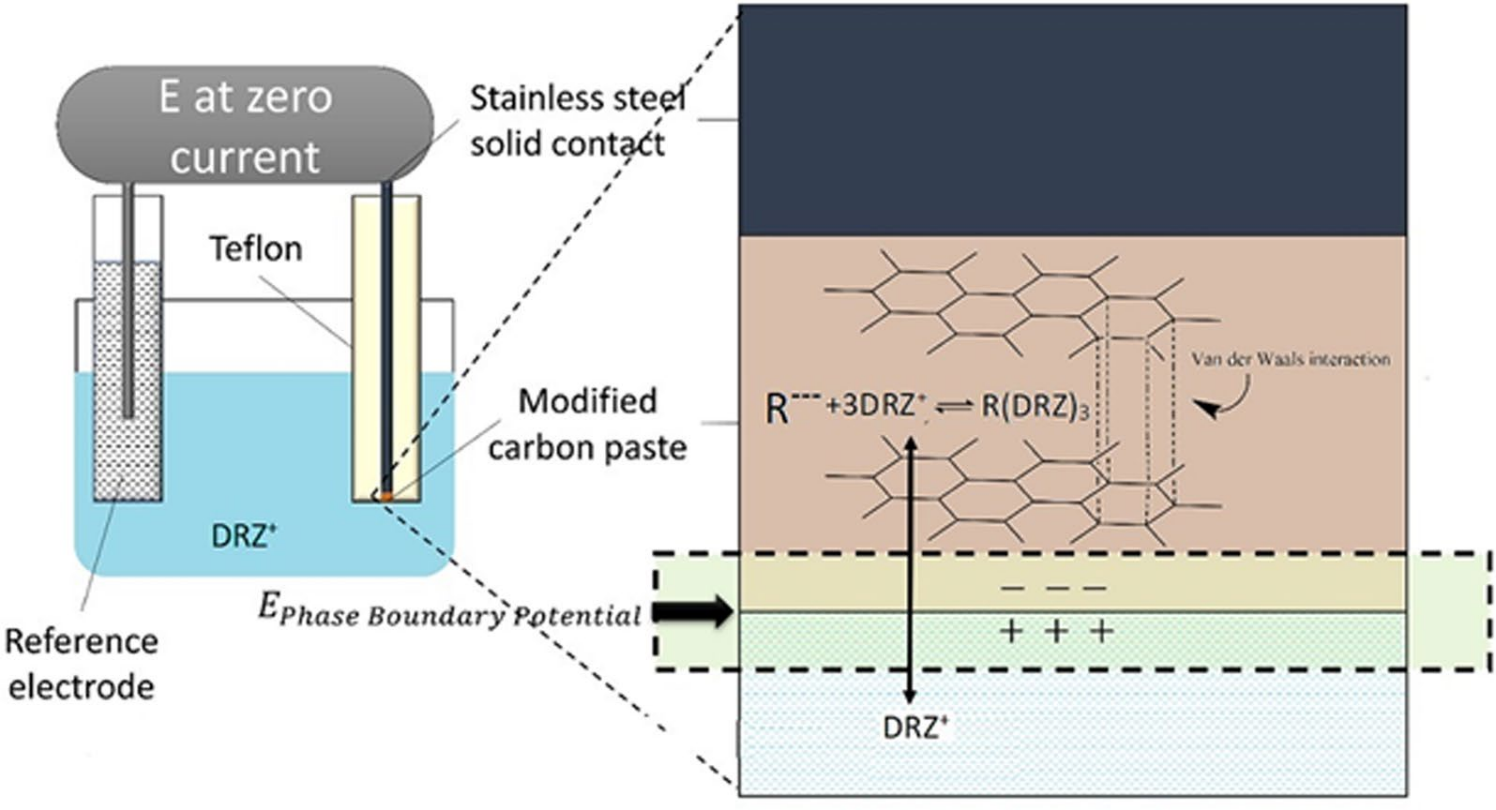
A schematic diagram of the mechanism involved in the DRZ ion detection

The impact of the ion exchanger amount on the potential response of the electrode was determined [Table 1], and it was found that the best result was achieved by increasing the amount of the ion exchanger to 20% [19]. In graphite, each carbon atom forms covalent bonds with three adjacent carbon atoms creating a layer of hexagonal arrays. The unbound carbon electrons, one from each carbon atom, collectively form an electron sea, which loosely binds the layers together through Van der Waals interactions [20]. Graphite allows the intercalation of the DRZ-PM complex between its layers. Charge transfer occurs between the complex and graphite to yield electrically conductive material in graphite intercalation compounds [21]. The plasticized carbon paste contains a DRZ-PM complex. The difference in DRZ activity between the carbon paste and the aqueous phase generates a force that drives DRZ to partition into the aqueous phase. Interfacial charge separation occurs as the positive DRZ ions cross the interface. In the absence of current, a phase boundary potential (EPBP) develops to counterbalance this driving force [22] (Fig. 3).

2 $$\text{EPBP}=\text{RT}/2\text{F} \,\text{ln}\, \text{kDRZ}^{1+}\text{aDRZ}^{1+}\,(\text{aqueous phase})/\text{aDRZ}^{1+} \,(\text{carbon paste})$$

In which DRZ^1+^ is the DRZ ion activity, R is the gas constant, T is the absolute temperature, F is the Faraday constant, and kDRZ^1+^ is a constant that includes the standard Gibb's free energy of DRZ ion transfer.

#### The plasticizer selection

**Fig. 4:**
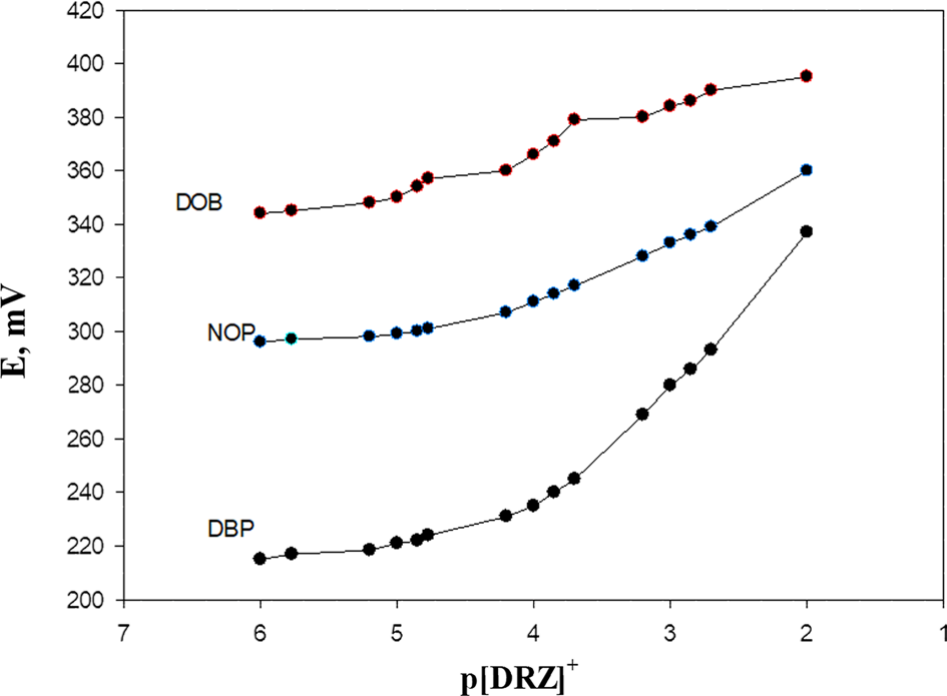
Impact of various levels of different plasticizers on DRZ-PM electrode response

The plasticizer must have a high capability to dissolve the substrate and other additives present. The nature of the plasticizer affects the polarity of the paste and the movement of ion-pair molecules [23, 24]. To evaluate the effect on the electrochemical behavior of the electrodes, various plasticizers such as DOP, *o*-NPOE, and DBP were used. It is necessary to determine the appropriate plasticizer and the optimal amount to be added to the electrode paste. DBP, as a plasticizer, produces a nearly Nernstian linear slope over a wide concentration range (Table 1, Fig. 4). This is attributed to the ability of DBP to extract dorzolamide ions from aqueous solutions to the organic paste phase. Among the various compositions studied, the electrode with 20% DRZ-PM ion exchanger, 40% DBP, 30% carbon, and 10% silica exhibited the best electrode performance. The repeatability was assessed by analyzing three concentration levels (2 × 10^–4^, 1 × 10^–3^ and 1 × 10^–2^ mol L^−1^) using the proposed electrode, in triplicates of each sample in a single run (Three successive calibration experiments). The intermediate precision was determined using the same solutions used in repeatability determination but in three separate runs.

The analysis gave satisfactory findings, and the percentage of the relative standard deviation (RSD) was less than 2% as shown in Table 2. Therefore, various operational characteristics of the electrodes were determined by applying this composition (Table 2). The electrochemical performing properties of the electrode were evaluated with the procedures of the IUPAC recommendations [25].

**Table 2:** Electrochemical response of the investigated DRZ-PM/silica selective electrode

Parameter	Value or range
Range	1.38 × 10^−4^–1 × 10^−2^ mol L^−1^
Linearity	
Slope (S)	59.00 ± 0.47
Correlation coefficient (r)	0.993
Intercept	49.5
Limit of detection (LOD)*	1 × 10^−4^ mol L^−1^
Working pH range	4.5–8.5
Response time (Rs)	4 s
Electrode life time	14 days
Accuracy** (mean ± SD)	99.46 ± 0.403
Precision (RSD%)	
Repeatability***	1.155
Intermediate precision****	1.298

*LOD (detection limit) was calculated by interception of the extrapolated lines of nonresponsive and the calibration plots for Nernstian ranges of Fig. 7

**Accuracy: DRZ HCL solution average recovery results (2 × 10^−4^, 1 × 10^−3^, 1 × 10^−2^ mol L^−1^) analysed in duplicate

***The intraday (n = 3), RSD% of three concentrations (2 × 10^–4^, 1 × 10^–3^&1 × 10-^2^ mol L^−1^) was repeated three times within the same day

****The interday (n = 3), RSD% of three concentrations (2 × 10^–4^, 1 × 10^–3^& 1 × 10-^2^ mol L^−1^) was repeated three times in three consecutive days

### pH effect on response functions

**Fig. 5:**
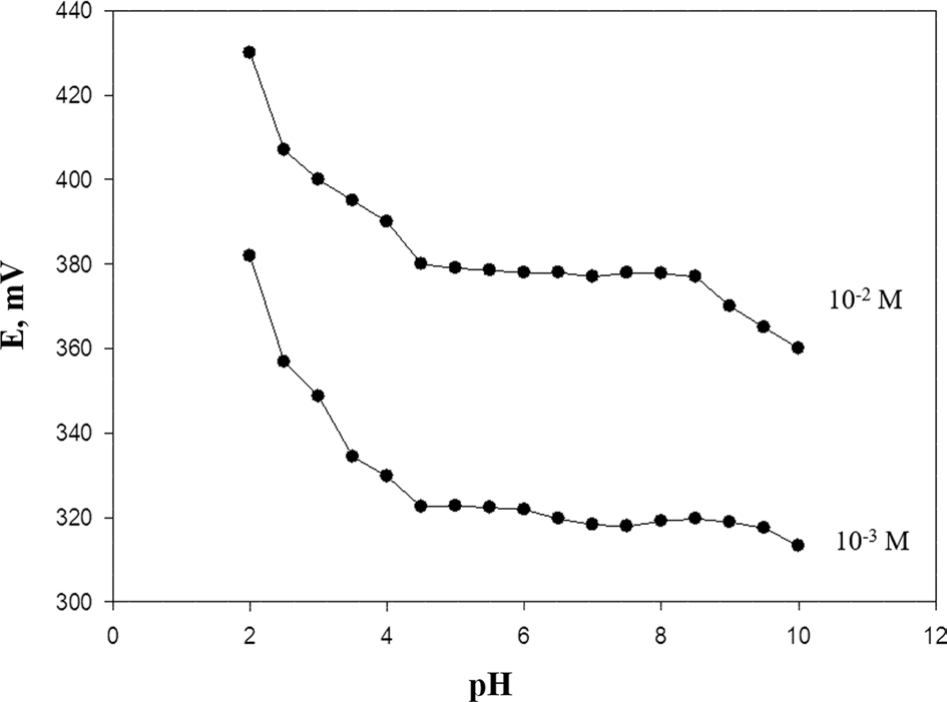
Impact of pH of measured DRZ solutions on the potential response of DRZ-PM/silica selective electrode

The determination and optimization of the pH effect on the constructed carbon paste DRZ-PM/silica electrode must be studied. The pH impact on their emf readings 1.0 × 10^−3^ mol L^−1^ DRZ test solution was determined firstly by 0.1^–1^ mol L^−1^ HCl and then the pH was raised gradually by 0.1–1 mol L^−1^ NaOH. The pH effect on the response of the ion selective electrode was determined for DRZ solutions over the concentration range (1 × 10^−3^, and 1 × 10^−2^ mol L^−1^), Fig. 5. The effective pH range of the fabricated electrodes to work suitably was 4.5–8.5. Although, there was a great increase in the potential at pH values lower than 4.5. Then, the potential was gradually reduced at pH values of more than 8.5 because of the increase of the unionized form of DRZ as it has a pKa value of 8.5.

### Selectivity

The selectivity of the constructed DRZ-PM/silica carbon paste electrode was evaluated in relation to various interferences, including cations such as K^+^, Na^+^, Cu^2+^, Ca^2+^, Fe^3+^ amino acids such as glycine and aminopencillic acid as well as the co-formulated drug, timolol maleate and the excipient benzalkonium chloride, as presented in Table 3. It was observed that the selectivity coefficient measured for the interfering ions and DRZ was less than 1, indicating that the DRZ-PM/silica carbon paste electrode exhibits a higher responsiveness towards DRZ.

**Table 3:** Selectivity coefficients (K^pot^_DRZ_^+^) For DRZ-PM/silica electrode by a separate solution method (10^−3^ mol·L^−1^ dorzolamide hydrochloride)

Interferent (1 × 10^−3^ mol L^−1^)	K^Pot^_DRZ_^+^
Na^+^	− 0.8377
K^+^	− 0.631
Ca^2+^	− 0.4368
Cu^2+^	− 0.5289
Fe^3+^	0.797
glycine	− 1.375
aminopencillic acid	− 1.059
Timolol maleate	− 0.311
Benzalkonium Cl	0.184

### Response time, repeatability, and life time of the proposed electrode including silica

**Fig. 6:**
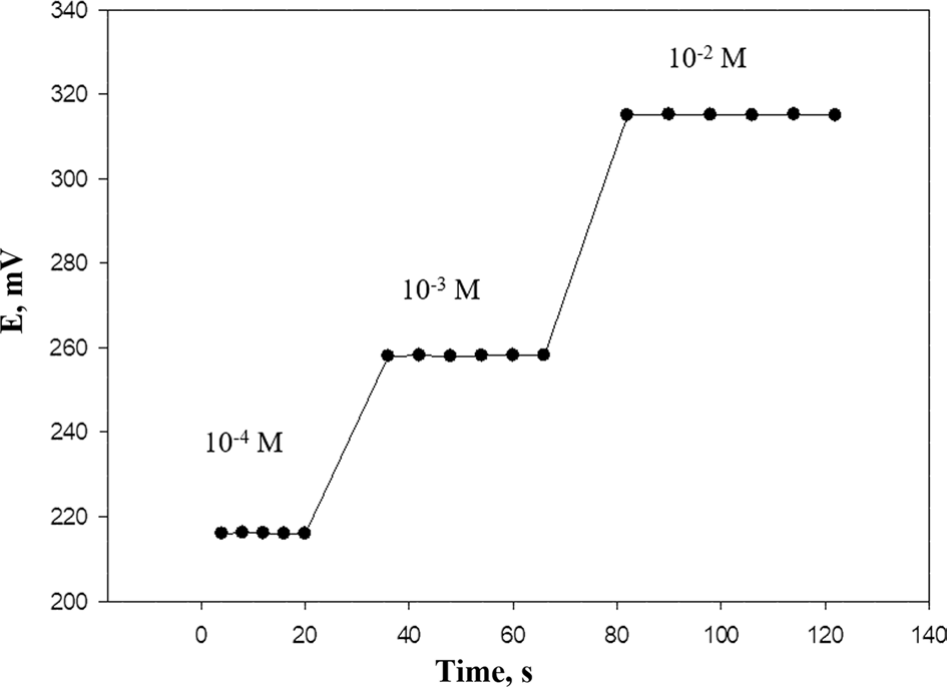
Typical potential–time plot for the DRZ-PM/silica electrode response

During the detection process of the selected drug using the DRZ-PM/silica carbon paste electrode, the response time was evaluated as an important characteristic. According to the IUPAC recommendations [25], the response time refers to the duration it takes for the potential reading of the electrode to reach its equilibrium value within 1 mV. In this study, the response time of each electrode was determined by varying the concentration of DRZ within the range of 1.0 × 10^–4^ to 1.0 × 10^–2^ mol L^−1^. The electrode achieved equilibrium in about 4 s and no change was observed up to 1 min (Fig. 6). To assess the potential reading repeatability, the electrode's response was measured by consecutively determining the DRZ concentration in a 1.0 × 10^–3^ mol L^−1^ of DRZ solution immediately after the determination of the first set of solutions in 1.0 × 10^−4^ mol L^−1^ of DRZ. The electrode potential for five replicate determinations applying a 1.0 × 10^–3^ mol L^−1^ of the DRZ solution gave an average potential of 258 (mV) and a standard deviation of ± 0.95. The corresponding values for 1.0 × 10^−2^ mol L^−1^ solution averaged 315 (mV) with a standard deviation of ± 0.75. Furthermore, the performance characteristics of the examined electrode were investigated in terms of soaking time where the electrode was immersed in 10^−3^mol L^−1^ solution of DRZ for different time intervals starting from 15 min until 14 days. The slopes of the electrode were determined and a gradual reduction in the potential after 14 days was observed so the optimum soaking time was 15 min.

### Analysis of pharmaceutical formulation by potentiometric titration and direct calibration curve methods

**Fig. 7:**
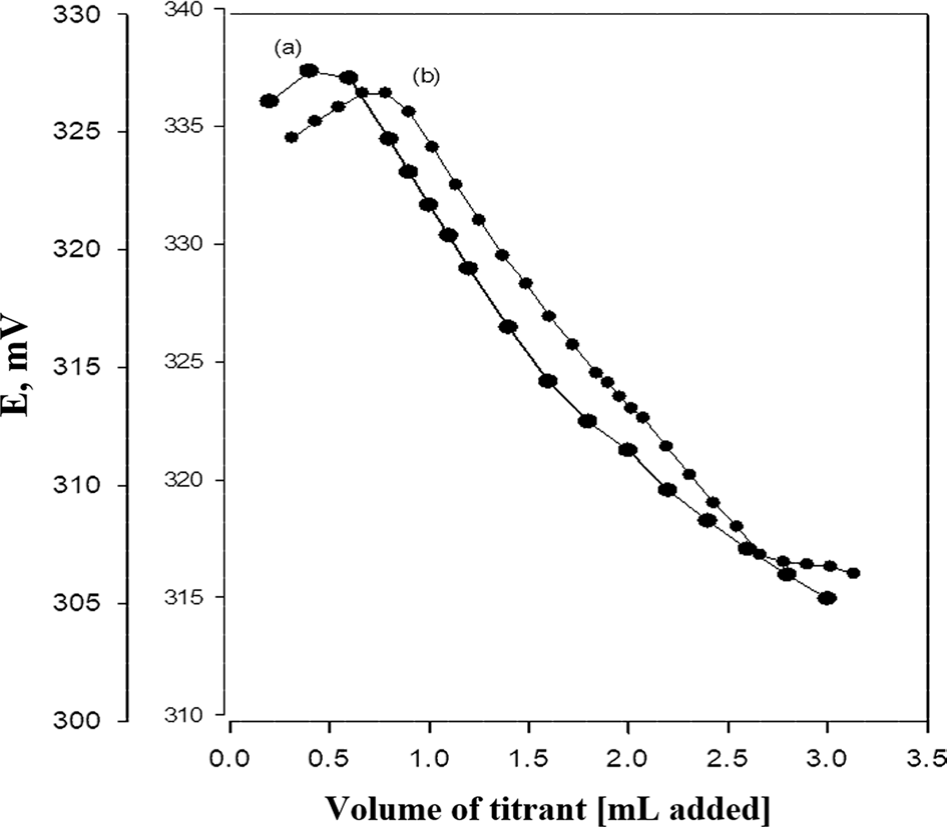
Potentiometric titration curve of **a** 3 mL and **b** 9 mL of 0.01 mol L^−1^ solution of DRZ with 0.0033 mol L^−1^ of PMA

The proposed DRZ-PM/silica electrode was effectively employed to analyze DRZ in ophthalmic dosage form using potentiometric titration and direct calibration curve techniques. The potentiometric titrations were conducted using 3 mL and 9 mL of 0.01 mol L^−1^ of DRZ with 0.0033 mol L^−1^ PMA solution, utilizing the DRZ-PM/silica electrode as a working electrode. The titration method depends on the reduction in the concentration of dorzolamide ion (DRZ +) via precipitation with a PM solution. By analyzing the inflection point of the titration curve obtained using the suggested sensor for the two concentrations in triplicate, the DRZ concentration could be accurately determined (Fig. 7). The results were expressed as percentage recoveries, which were found to be 100 ± 0.2 and 100.32 ± 0.14 for 3 mL and 9 mL that were taken from 1 × 10^–2^ mol L^−1^ DRZ, respectively as in Table 4.

**Table 4:** DRZ determination in Xolamol® eye drops using the proposed DRZ-PM/silica electrode

Pharmaceutical formulation	Applied method	Taken conc (mol L^−1^)	Found conc (mol L^−1^)	Recovery% ± RSD%
Xolamol eye drop 22.26 mg DRZ HCl/mL Batch number(VB0113)	Potentiometric titration method	1.00 × 10^–2 *^ 1.00 × 10^–2**^	1.00 × 10^–2^ 1.0032 × 10^–2^	100.00 ± 0.20 100.32 ± 0.14
	Calibration curve	2.00 × 10^–4^	1.995 × 10^–4^	99.75 ± 0.10
		1.00 × 10^–3^	0.999 × 10^–3^	99.90 ± 0.18
		1.00 × 10^–2^	0.9985 × 10^–2^	99.85 ± 0.102

*3 mL was taken of 1 × 10^−2^ mol L^−1^ DRZ

**9 mL was taken of 1 × 10^−2^ mol L^−1^ DRZ

**Fig. 8:**
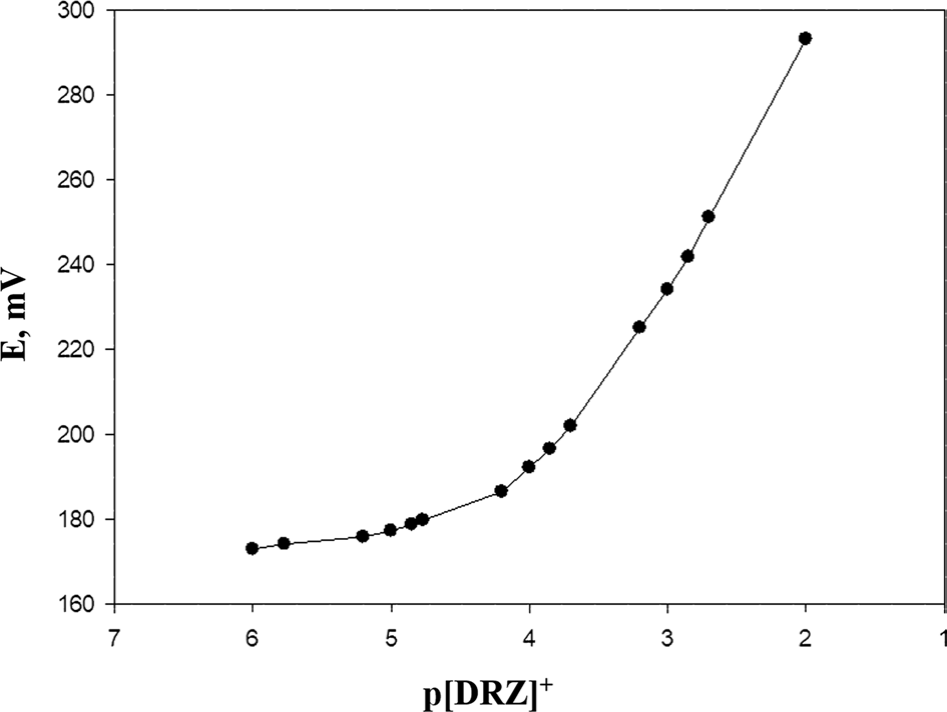
Calibration curve of DRZ-PM/silica electrode

The calibration curve of the DRZ ion was plotted in the concentration range of 1.0 × 10^–6^ to 1.0 × 10^–2^ mol L^−1^ and the estimated emf was observed applying DRZ-PM inclusion silica electrode for the three concentrations in duplicate (Fig. 8).

**Fig. 9:**
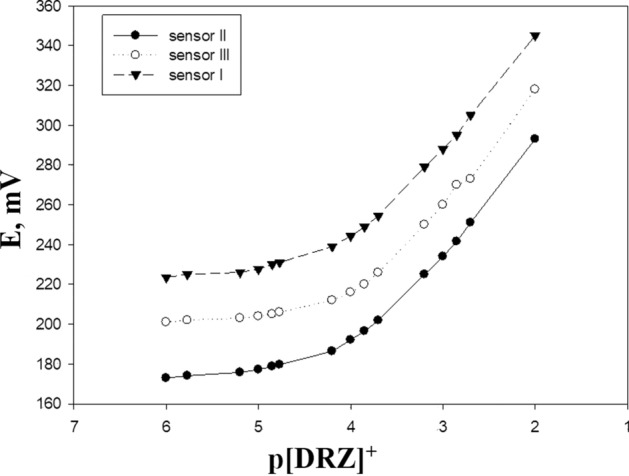
Calibration curves of the three fabricated sensors

Data was plotted using the recorded emf versus the log of the DRZ + concentration and the resulting curve was used for the measurement of unknown DRZ + concentrations. The obtained results were determined as the % recoveries of 99.75 ± 0.1, 99.9 ± 0.18, and 99.85 ± 0.102 for concentrations of 2 × 10^−4^, 1 × 10^−3^, 1 × 10^−2^ (mol L^−1^), respectively (Table 4). While Fig. 9 showed the three calibration curves of the three fabricated sensors.

F-test and Student t-test were conducted to compare the accuracy achieved by the proposed method with a previously reported electrochemical method for both drug substance and ophthalmic dosage form analysis [26]. The calculated F-test and t-test values, as presented in Tables 5 and 6, were found to be lower than the critical values. This indicates that there is no significant difference between the accuracy and precision of the two techniques at a 95% confidence level.

**Table 5:** Statistical analysis of the proposed methods to assess dorzolamide HCl in its drug substance using modified DRZ-PM/silica electrode

Parameters	Proposed methods	Reported method (26)
Mean Recovery%	99.46 ± 0.40	99.81 ± 0.20
n	6	7
SD	0.40	0.20
Variance	0.162	0.064
Student t-test	1.84(2.201)	–
F-Test	2.5 (4.93)	–

Confidence limit at 95%, SD standard deviation, n number of determinations, values between parenthesises are the critical values of Student t-test and F-value

**Table 6:** Statistical analysis of the proposed methods to evaluate dorzolamide HCl in its ophthalmic dosage form using modified DRZ-PM/silica electrode

Parameters	Proposed methods		Reported method (26)
	Calibration curve	Potentiometric titration	
Mean recovery	99.83 ± 0.38	100.1667 ± 0.34	99.82%
n	6	6	7
SD	0.38	0.34	0.205
Variance	0.146	0.118	0.042
Student t-test	0.058 (2.201)	2.15(2.201)	
F-Test	3.47 (4.93)	2.8 (4.93)	

Confidence limit at 95%, SD standard deviation, n number of determinations, values between parenthesises are the critical values of Student t-test and F-value

## Conclusion

In this study, dorzolamide-phosphomolybdic acid was used as the electro-active compound, and three electrodes for the measurement of DRZ were constructed. The three constructed electrodes were used as conventional carbon paste (electrode I), while electrodes II and III were modified by applying silica and β-cyclodextrin might be a valuable analytical mechanism and an interesting substitute for the measurement of dorzolamide HCl in its" API" and ophthalmic preparations. The electrodes have great sensitivity, excellent selectivity, a fast response, and a wide range of concentration. The results obtained stated that the sensitivity and selectivity of electrode III were enhanced more than electrode I because of using of β-cyclodextrin as a modifier, while, by using silica in the electrode a great sensitivity and reproducibility for the determination of the dorzolamide were recorded.

## Data Availability

Data will be made available on request.

## References

[1] Martens-Lobenhoffer J, Banditt P (2002). Clinical pharmacokinetics of dorzolamide. Clin Pharmacokinet.

[2] Balfour JA, Wilde MI (1997). Dorzolamide: a review of its pharmacology and therapeutic potential in the management of glaucoma and ocular hypertension. Drugs Aging.

[3] Stani Z, Girousi S. Carbon paste electrodes in potentiometry: the state of the art and applications in modern electroanalysis pp. 89128. © 2011 University Press Centre, Pardubice, Czech Republic. ISBN 978-80-7395-434-5 (printed); 978-80-7395-435-2 (online)

[4] Shao Y, Ying Y, Ping J (2020). Recent advances in solid-contact ion-selective electrodes: functional materials, transduction mechanisms, and development trend. Chem Soc Rev.

[5] Erk N (2002). Simultaneous determination of dorzolamide HCl and timolol maleate in eye drops by two different spectroscopic methods. J Pharm Biomed Anal.

[6] Sharath HM, Jose GB, Channabasavaraj KP, Modiya JS (2011). Development and validation of spectrophotometric methods for estimation of dorzolamide HCl in bulk and pharmaceutical dosage forms. Int J Pharm Sci Res.

[7] Dias AN, Subrahmanyam EVS, Shabaraya R, Misquith J (2013). New analytical method and its validation for the estimation of dorzolamide hydrochloride in bulk and marketed formulation. Int J Chem Tech Res.

[8] Deshpande SV, Funne SM, Mahaparale SP, Onkar PR (2014). Development and validation of UV spectrophotometric method for estimation of timolol maleate and dorzolamide hydrochloride in bulk and eye drop formulation. Int J Pharm Chem Sci.

[9] Erk N (2003). Voltammetric and HPLC determination of dorzolamide hydrochloride in eye drops. Pharmazie.

[10] Kanchan RU, Shikha MNR, Rane RB (2008). Simultaneous RP-HPLC determination of dorzolamide hydrochloride and timolol maleate in pharmaceutical preparations. Anal Chem Ind J.

[11] Shadoul WA, Gad-Kariem EA, Adam ME, Ibrahim KEE (2011). Simultaneous determination of dorzolamide hydrochloride and timolol maleate in ophthalmic solutions using HPLC. Elixir Pharm.

[12] Nagoria BP, Maru A, Muysunic P, Guptad S (2011). Method development and its validation for simultaneous estimation of timolol maleate and dorzolamide hydrochloride in as API and in ophthalmic solution dosage form by RP-HPLC. J Chem Pharm Res.

[13] Kumar SR, Charumathi S, Lavanya J, Duganath N, Kumar PBR, Devanna N (2013). Development and validation of RP-HPLC method for dorzolamide hydrochloride in bulk and pharmaceutical dosage form. Am J Pharm Tech Res.

[14] Bebawy LI (2002). Application of TLC-densitometry, first-derivative UV-spectrophotometry and ratio derivative spectrophotometry for the determination of dorzolamide hydrochloride and timolol maleate. J Pharm Biomed Anal.

[15] Singh R, Bharti N, Madan J, Hiremath SN (2010). Characterization of cyclodextrin inclusion complexes. J Pharm Sci Technol.

[16] Umezawa Y, Buhlmann P, Umezawa K, Tohda K, Amemiya S (2000). Potentiometric selectivity coefficients of ion-selective electrodes. Pure Appl Chem.

[17] Ibrahim H, Issa MY, Abu-Shawish MH (2007). Improving the detection limits of antispasmodic drugs electrodes by using modified membrane sensors with inner soli contact. J Pharm Biomed Anal.

[18] Masadome T, Yang J, Imato T (2004). Effect of plasticizer on the performance of the surfactant-selective electrode based on a poly (vinyl chloride) membrane with no added ion-exchanger. Micro chim Acta.

[19] Arvand M, Asadollahzadeh AS (2008). Ion-selective electrode for aluminum determination in pharmaceutical substances, tea leaves, and water samples. Talanta.

[20] Tanzi MC, Farè S, Candiani G (2019). Foundations of biomaterials engineering.

[21] Rahaman M, Nayak L, Hussein IA, Das NC (2021). Polymer nanocomposites containing graphene.

[22] Bakker E, Pretsch E (2002). Peer reviewed: the new wave of ion-selective electrodes. Anal Chem.

[23] Egorov VV, Bolotin AA (2006). Ion-selective electrodes for the determination of organic ammonium ions: ways for selectivity control. Talanta.

[24] Zhang BX, Han XZ, Fang HZ, Shen LG, Yu QR (2006). 5,10,15 Tris(pentafluorophenyl)corrole as a highly selective neutral carrier for a silver ion-sensitive electrode. Anal Chim Acta.

[25] Buck RP (1994). ELindner: IUPAC recommendation for nomenclature of ion selective electrodes, pure and applied. Chemistry.

[26] Alarfaj NA, El-Tohamy MF (2016). New electrochemically-modified carbon paste inclusion β-cyclodextrin and carbon nanotubes sensors for quantification of dorzolamide hydrochloride Int. Int J Mol Sci.

